# Composite Materials Used for Dental Fillings

**DOI:** 10.3390/ma17194936

**Published:** 2024-10-09

**Authors:** Joanna Wysokińska-Miszczuk, Katarzyna Piotrowska, Michał Paulo, Monika Madej

**Affiliations:** 1 Faculty of Medical Dentistry, Medical University of Lublin, ul. Chodźki 6 Ave, 20-093 Lublin, Poland; joanna.wysokinska-miszczuk@umlub.pl (J.W.-M.); michal.paulo@umlub.pl (M.P.); 2 Faculty of Mechatronics and Mechanical Engineering, Kielce University of Technology, al. Tysiąclecia Państwa Polskiego 7, 25-314 Kielce, Poland; mmadej@tu.kielce.pl

**Keywords:** dental composites, ormocer resin, surface texture, hardness, wettability, friction, wear

## Abstract

This article explores the properties of composite materials employed in dental fillings. A traditional nano-hybrid composite containing nanofiller particles exceeding 82% by weight served as a benchmark. The remaining samples were fabricated from ormocer resin, maintaining an identical nanofiller content of 84%. In all specimens, the nanoparticles were dispersed randomly within the matrix. This study presents findings from investigations into surface geometry, hardness, wettability, and tribological behavior. The microscopic observations revealed that ormocer-based samples exhibited greater surface roughness than those composed of the traditional composite. Hardness testing indicated that both ceramic addition and sample preparation significantly influenced mechanical properties. Ceramic-enhanced samples demonstrated superior hardness, surpassing the reference composite by 30% and 43%, respectively. Contact angle measurements revealed hydrophilic characteristics in the classic composite, contrasting with the hydrophobic nature of ceramic-containing samples. Tribological evaluations revealed the superiority of the classic composite in terms of friction coefficients and volumetric wear compared to ormocer-based materials.

## 1. Introduction

Modern dentistry offers a range of advanced materials capable of restoring both the function and esthetics of teeth, such as the shape and color of damaged tissues. These solutions are tailored to the individual needs and expectations of patients, and dental services not only provide therapeutic benefits but also enhance the quality of life [1]. Materials used for dental fillings must possess suitable mechanical, tribological, and physicochemical properties while maintaining biocompatibility with natural tooth tissues.

When designing new dental composites, it is crucial to achieve composites with a low coefficient of friction and minimal wear while exerting minimal influence on the wear of opposing tooth tissues. To reduce wear and the coefficient of friction of dental materials, the composition of the fillers used is intensively modified [2]. The latest literature indicates a significant impact of nanoparticles on the structure and performance properties of composites. Compared to commonly used macro- and microparticles, nanoparticles are characterized by uniform dispersion in the resin on a nanoscale. As a result, nanocomposites exhibit greater hardness and wear resistance. Additionally, the presence of nanoparticles contributes to a reduction in polymerization shrinkage, a decrease in water absorption, and an increase in the likelihood of achieving a smoother surface due to polishing [3,4].

A key aspect of designing new dental composites is achieving materials with low friction coefficients and wear resistance, ensuring longer service life and minimal impact on the wear of opposing tooth tissues. Dental composites should exhibit a synergistic interaction with natural tooth tissues. To improve these properties, the composition of the fillers used is continuously modified, with the use of nanoparticles playing an increasingly important role. Nanoparticles, due to their uniform distribution in the resin matrix, significantly improve the hardness, wear resistance, and esthetic properties of composites. This distribution, on a nanoscale, allows for the creation of a more homogeneous structure, which reduces stress concentration and improves the strength of the material. The large specific surface area of nanoparticles enables better bonding with the matrix, increasing adhesion between the components of the composite. The use of nanoparticles also improves the esthetic properties of dental composites, as they have the ability to scatter light in a manner closer to that of natural tooth tissue. When reconstructing teeth in visible areas, using nanocomposites allows for better color matching and translucency. Additionally, the use of nanocomposites increases durability and reduces the risk of material degradation, as they exhibit higher resistance to chemical agents present in the oral cavity, such as acids and enzymes. All these aspects are crucial for the long-term success of dental treatment. Modifying dental composite fillers by introducing nanoparticles into the polymer matrix represents a modern approach to improving the quality and effectiveness of materials used in dentistry [4,5,6].

The literature features extensive research on nanofiller-reinforced dental composites [7,8,9,10,11], yet the diversity of components complicates direct comparisons of their performance characteristics.

The researchers in [12] examined how adding silica nanoparticles to ceramic–polymer composites affected their mechanical and wear-and-tear properties. They focused on how the type, amount, and size of the filler particles influenced the overall performance of the composite. The researchers primarily used silica powder with particles 5 ÷ 10 μm in diameter and a density of 2.38 g/cm^3^. They incorporated a nanofiller, silanized silica R709, with particles measuring 40 nm and a density of 2.20 g/cm^3^. The organic component consisted of methacrylate monomers: Bis-GMA and TEGMA. The filler constituted 55–60% of the composite’s volume. The prepared samples were solidified using a light-activated polymerization process.

Vickers microhardness testing revealed that adding nanofillers enhanced the composite’s hardness. Samples composed entirely of micro-silica powder, with 60.7% filler, exhibited a hardness of 58.8 kPa. However, when 15% of the filler was replaced with nanopowder, the hardness increased to 65.5 kPa, exceeding the original hardness by over 10%. Wear-and-tear evaluations demonstrated that samples containing both micro- and nano-silica powders, with a total filler content of 60% and 10% nanopowder, exhibited the lowest wear. Furthermore, the friction coefficients were lowest when 10% of the filler consisted of nanopowder. According to these authors, the addition of SiO nanopowder has a positive impact on microhardness and tribological properties, with the best results obtained at 10–15% volume of the composite.

In [13], the authors also investigated the tribological properties of ceramic–polymer composites. The composites had an organic matrix comprising a mixture of Bis-GMA, TEGDMA, and DEA-EMA resins, and a system of photoinitiators and stabilizers. The authors introduced powdered fillers such as fluoride, nanosilica (n-SiO_2_), and a friction modifier—polyethylene (PE)—into the organic matrix. The fillers underwent a silanization process in a silane solution. This process involved depositing active silane groups onto the surface of the filler particles in a vacuum evaporator. After homogenization, the composites were cured in PTFE molds for 40 s. Friction tests were conducted on a specially designed dental friction simulator—a pneumatically controlled pin-on-disc tribological tester operating in reciprocating motion under lubrication with a pH 6.8 solution, corresponding to the pH of human saliva. The counter sample was human enamel embedded in an aluminum frame. The results showed that both the strontium fluoride-based composite and the ytterbium fluoride-added composite exhibited similar friction coefficient values, with slightly lower values recorded for the composite with added ytterbium fluoride. Simultaneously, greater linear and volumetric wear was recorded for this material. During the study, the authors observed that the wear of ceramic–polymer composites intended for permanent dental fillings depended on the type of powdered filler and was lower for a composite with strontium fluoride.

Studies [14,15,16,17,18,19,20,21] indicate that the tribological properties of dental resin-based composites are significantly influenced by the morphology, homogeneity, and concentration of inorganic fillers. The authors of [16] investigated the effect of hydroxyapatite (HA) particles on the tribological behavior of resin-based composites. They found that a composite containing 0.4% by volume of filler particles exhibited optimal hardness, reduced wear, and a lower coefficient of friction. In addition to HA particles, [17] explored the effect of reinforcing the polymer matrix with two distinct types of ceramic particles, alumina and silica. The authors concluded that incorporating alumina into the polymer matrix enhances wear resistance. In turn, [18] observed that composites with organic fillers demonstrated the lowest coefficient of friction and wear.

Recent studies have dealt with the application of nanomaterials to different medicine areas, which has led to a new discipline known as nanomedicine. Nanotechnology has the potential to revolutionize the field of healthcare diagnostics by improving the accuracy, sensitivity, and speed of medical tests. Polymer nanocomposites offer new opportunities for modern medicine to generate products for antibacterial treatment [22], tissue engineering [23], cancer therapy [24], medical imaging [25], dental applications [26], drug delivery, etc. [27]. There are also potential uses in designing medical tools and processes for the new generation of medical scientists.

This study comprehensively characterized the surface layer and mechanical/physicochemical properties of materials used for dental fillings. A key aspect was evaluating their tribological behavior when lubricated with artificial saliva. The innovative approach involved applying materials in single or multiple layers and conducting tests at 37 °C to mimic oral conditions.

## 2. Materials and Methods

The NHO-1 and NHO-4 test materials were made of ormocer resin. These materials have indications for single-layer restorations up to 4 mm. They consisted of nanoscale particles and did not differ in filler content, with the filler particles being randomly dispersed in the matrix (in all tested composites). The NH sample, on the other hand, served as a reference material with a nano-hybrid structure containing nanoparticle fillers. Samples were prepared according to the manufacturer’s recommendations. The characteristics of the composite resins used are presented in Table 1.

For NHO-1 and NHO-4 (VOCO, Cuxhaven, Germany), each 0.5 mm thick layer was applied directly from the package and then condensed using dental instruments (a flat plastic tool) for 60 s. A flat plastic tool is a basic dental instrument used for applying and modeling fillings. It has two tips: a spatula and a ball. The spatula is suitable for applying the material, modeling the cusps, and giving the filling its final shape. The ball end (a ball burnisher) works perfectly when condensing the material. The first layer was applied directly to the surface of the metal piston, and then, using an LED Translux Wave lamp (KULZER, Hanau, Germany) with a power of >1200 mW/cm^2^ and a wavelength of 440–480 nm, the first 0.5 mm thick layer was irradiated for 60 s. Subsequent portions of the composite were applied to the already polymerized layers and irradiated again. For each irradiation, the SoftStart function was used (this involves starting the polymerization with a relatively low-intensity light, which gradually increases during the device’s operating cycle). Before each use of the lamp, its power was checked using the measuring device installed in the lamp base. Samples were produced using the composite at room temperature. The NH material (Megadenta, Radeberg, Germany) was prepared as a single 3 mm thick layer. It was applied in a single portion directly to the device piston and then condensed using dental instruments for 120 s (a flat dental condenser and a ball condenser). The thickness of a single layer was permissible by the manufacturer according to the instructions (a layer up to 4 mm thick). Then, using an LED Translux Wave lamp with a power of >1200 mW/cm^2^ and a wavelength of 440–480 nm, the entire 3 mm thick layer was irradiated for 60 s.

**Figure 1:**
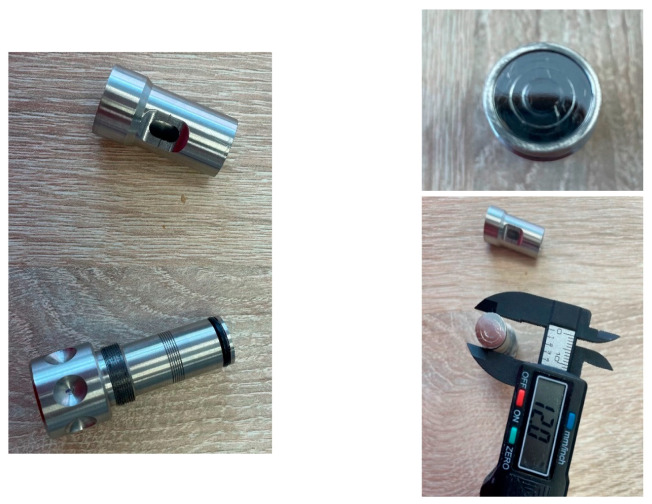
A device for creating custom composite samples.

The samples were prepared using a device (Figure 1) designed for creating individual color guides for composite or porcelain materials. This tool enabled the production of discs with a diameter of 12 mm and a thickness of 3 mm. Thanks to the design of this tool and the embedded scale, it was possible to precisely measure the thickness of each individual layer applied to create the final sample in the form of a disc.

A single operator prepared the test materials and avoided using additional dental equipment for polishing (Smile Line USA Inc., Wheat Ridge, CO, USA) after polymerization. The final layer was condensed and smoothed with a dental flat plastic tool to achieve smooth surfaces. The geometric structure of the surfaces before the tribological tests was observed using a DCM8 confocal microscope (Leica, Geneva, Switzerland). Surface topography analysis was performed based on 3D axonometric images, surface profiles, material composition curves, and selected amplitude parameters (Sq, Sv, Sp, Ssk, and Sku). The surface area under analysis was 0.157 mm^2^. The results of the experiments are presented in Section 3.1.

To determine the mechanical properties, an ultra-nanoindentation hardness tester (UNHT) (Anton Paar, Baden, Switzerland) and a Berkovich indenter were employed in the instrumental indentation method. Hardness, Young’s modulus, plastic work, and elastic work were calculated from the load–displacement curve. A constant loading and unloading rate of 100 mN/min, a maximum load of 50 mN, and a 5 s hold were used. The results of these tests are presented in Section 3.2.

Wettability was assessed using an Attention Theta (Biolin Scientific, Tietäjäntie, Finland) optical tensiometer and the sessile drop method. A 4 µL droplet of demineralized water and artificial saliva solution was placed on the sample surface, and the contact angle was immediately measured. Average angle values were determined from five sets of measurements. The tests were conducted at a temperature of 23 ± 1 °C and 55 ± 5% humidity. The results are presented in Section 3.3.

**Figure 2:**
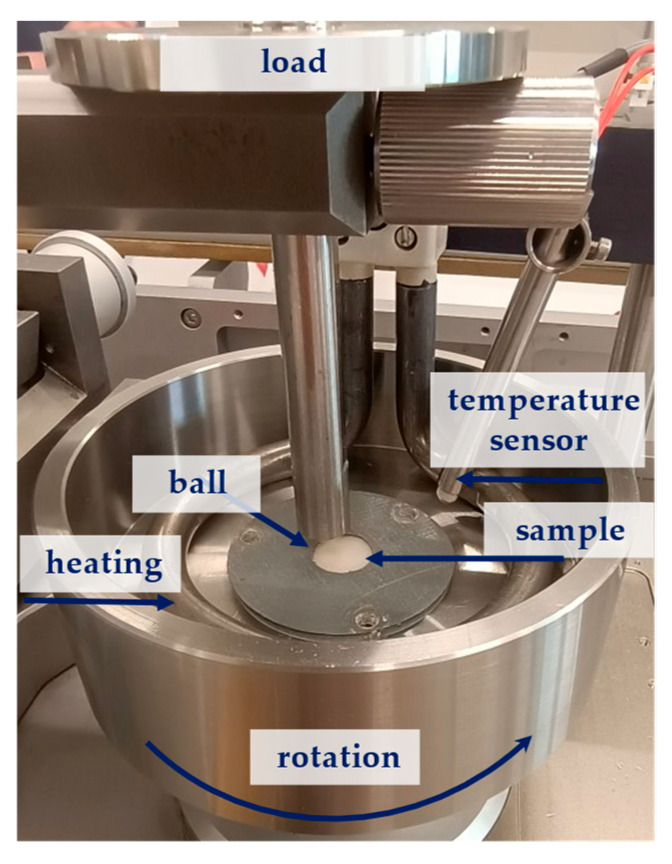
Friction pair.

Tribological tests were performed using an TRB3 tribometer (Anton Paar, Baden, Switzerland). The tests were conducted in reciprocating motion under simulated saliva lubrication at 37 °C. The test parameters included a load of 1 N, frequency of 1 Hz, stroke length of 3 mm, angle of 60°, and 10,000 cycles of friction. A schematic of the friction junction is presented in Figure 2. The results are presented in Section 3.4.

The counter sample in the tested friction junctions was a 6 mm diameter ZrO_2_ ball (Table 2).

The chemical composition of the lubricant is presented in Table 3.

Following tribological testing, the samples were subjected to microscopic observations. The measurement area for each sample was 800 by 1000 μm. The study determined the volumetric wear of the wear tracks. To complement the study, amplitude parameters within the wear tracks were determined. The parameters were analyzed on areas of 150 by 200 μm and compared to values outside the wear track. The results are presented in Section 3.5.

## 3. Results

### 3.1. Confocal Microscopy Results

**Figure 3:**
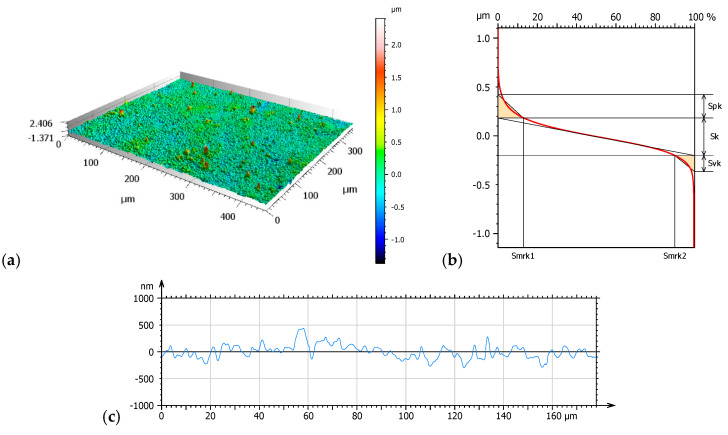
NH—axonometric image 3D (**a**), material ratio curve (**b**), primary surface profile (**c**).

**Figure 4:**
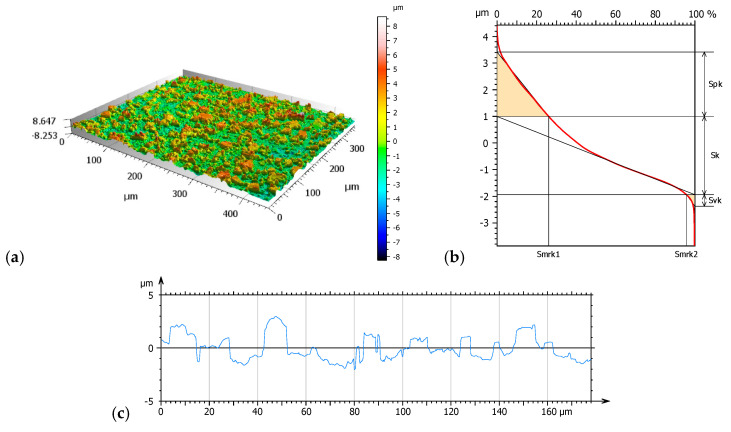
NHO-1—axonometric image 3D (**a**), material ratio curve (**b**), primary surface profile (**c**).

**Figure 5:**
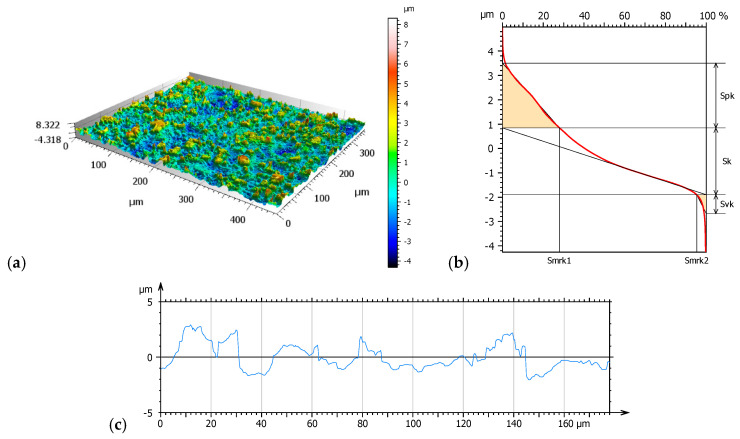
NHO-4—axonometric image 3D (**a**), material ratio curve (**b**), primary surface profile (**c**).

**Figure 6:**
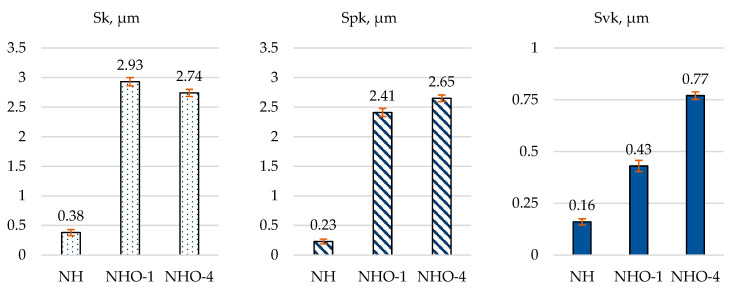
Core roughness parameters.

Figure 3, Figure 4 and Figure 5 present 3D isometric views (a), material ratio curves (b), and mean primary profiles (c). Additionally, core roughness parameters were determined based on the material ratio curves: Sk—core roughness depth, Spk—mean peak height above the core roughness level, and Svk—mean valley depth below the core roughness profile (Figure 6).

Amplitude parameters of the mean profile were determined to supplement the study. The results are shown in Table 4.

Geometric surface texture analysis revealed that samples NHO-1 and NHO-4 exhibited the most pronounced surface topography. This is evidenced by the values of the amplitude parameters: Sq, Sv, and Sp. The positive values of the skewness parameter (Ssk) for all tested samples indicate the presence of steep peaks with sharp crests, most notably in samples NHO-1 and NHO-4. The kurtosis value (Sku), a measure of the peakedness of the height distribution, is also sensitive to isolated peaks or valleys. A value close to 3 indicates a normal height distribution—an even distribution of peaks and valleys on the surface. Samples NHO-1 and NHO-4 exhibit such a distribution.

Analysis of the material ratio curves (Figure 6) suggests that samples NHO-1 and NHO-4 will exhibit both the longest running-in period and the best lubrication due to the highest values of the Svk and Spk parameters compared to the classical composite. The values of these parameters were more than 10 times higher than the classical composite. Additionally, for both samples, a higher friction force is expected in the initial phase of the tribological test.

### 3.2. Hardness

**Figure 7:**
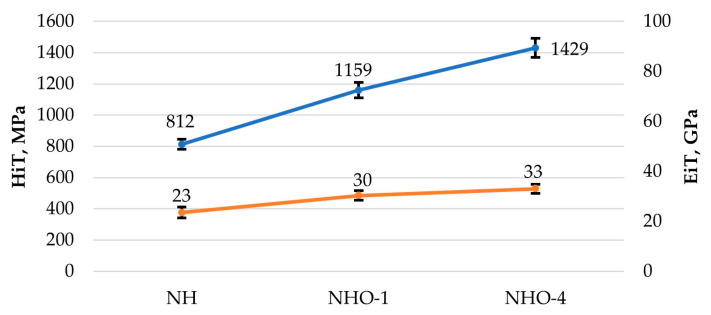
Results of mechanical tests: instrumental hardness (HIT), Young’s modulus (EIT).

Figure 7 shows the average values of the mechanical parameters, instrumental hardness (HIT) and Young’s modulus (EIT), calculated from 10 measurement series.

The hardness testing results indicated that the reference sample (NH) exhibited the lowest instrumental hardness of 812 MPa and a Young’s modulus of 23 GPa. These parameters were approximately 47% and 30% lower, respectively, compared to the values obtained for sample number 3.

Sample NH was a nano-hybrid material (possessing typical micro-hybrid properties with the advantages of nanotechnology) with a filler content >82% by weight. As a classic composite material without ceramic additives, it exhibited the poorest performance. Additionally, its polymerization shrinkage coefficient was <1.9%, while for samples NHO-1 and NHO-4 it was <1.25%. Samples NHO-1 and NHO-4 were ceramic-based materials, with both the filler and the composite matrix made of silicon dioxide. The filler volume fraction was 84%. These properties were responsible for achieving better results. The difference between samples NHO-1 and NHO-4 is related to the sample preparation method. Disk NHO-4 was made of six layers, each 0.5 mm thick. In contrast, disk NHO-1 was made from a single 3 mm thick layer that was then polymerized as a whole, unlike sample NHO-4, where each layer was polymerized separately. Preparing the disk from multiple layers of material reduced polymerization shrinkage and improved the material’s strength properties [30,31].

### 3.3. Contact Angle

**Figure 8:**
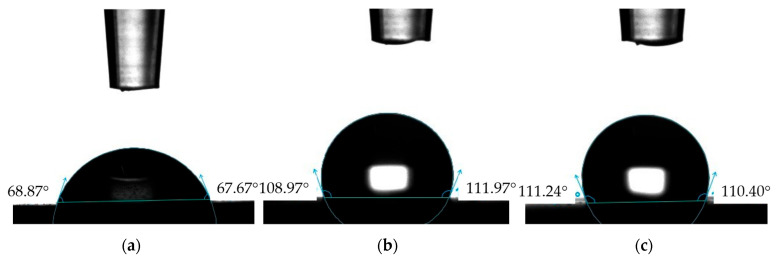
Examples of demineralized water droplets: (**a**) NH, (**b**) NHO-1, (**c**) NHO-4.

**Figure 9:**
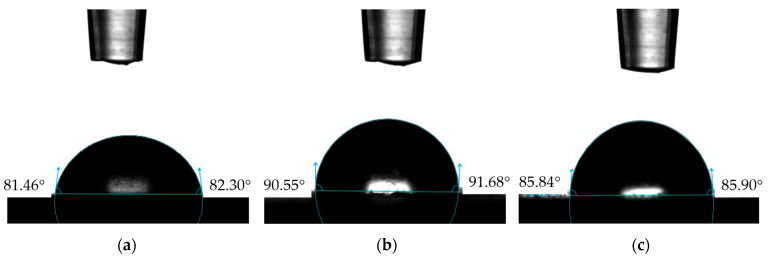
Examples of artificial saliva droplets: (**a**) NH, (**b**) NHO-1, (**c**) NHO-4.

**Figure 10:**
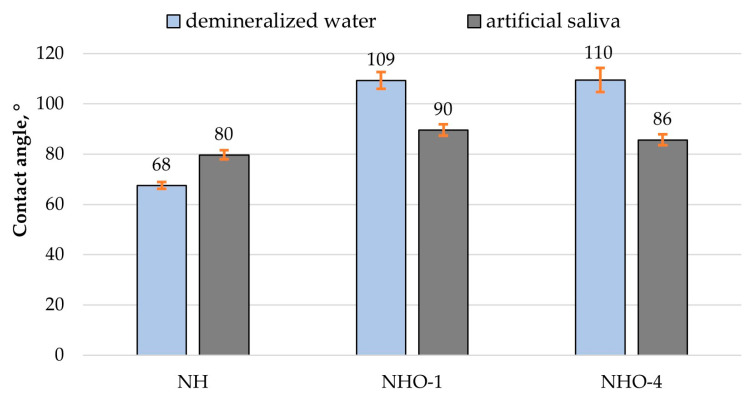
Average contact angle.

The contact angle plays a significant role in the performance of tribological systems. Figure 8 and Figure 9 show example images of demineralized water droplets deposited on the surfaces of the analyzed materials, while Figure 10 presents the average values of the recorded contact angles.

The results presented in Figure 10 indicate that the nature of the surface topography influences the contact angle values. The lowest contact angle values for demineralized water and artificial saliva were recorded for the reference sample NH, which had the least developed surface, while the highest values were for sample NHO-1, which had the highest roughness. Sample NH was characterized by good wettability and exhibited hydrophilic properties. In the case of samples NHO-1 and NHO-4, the contact angles with demineralized water were 110° and 109°, respectively, indicating their hydrophobic properties.

In a clinical context, dental fillings should exhibit hydrophobic properties [32]. Within the oral cavity, every tooth surface, as well as dental fillings used to restore tooth shape and function, comes into contact with saliva. Dental caries is an infectious disease, and bacteria migrate and settle on the surfaces of teeth and dental restorations. Increased hydrophilicity of dental materials can accelerate the degradation of bonds, thereby increasing the likelihood of microleakage between the patient’s tissues and the composite filling. This is particularly dangerous in areas that are poorly cleaned by the patient, such as class II and V restorations. In such cases, the risk of secondary caries is significantly increased. With increasing hydrophilicity of composite materials, there is an increased risk of discoloration at the interface between the materials and the patient’s tissues, as well as on the fillings themselves, which directly translates into a deterioration of the esthetic effect and increased patient dissatisfaction with treatment [33,34,35,36,37,38].

### 3.4. Tribological Tests

**Figure 11:**
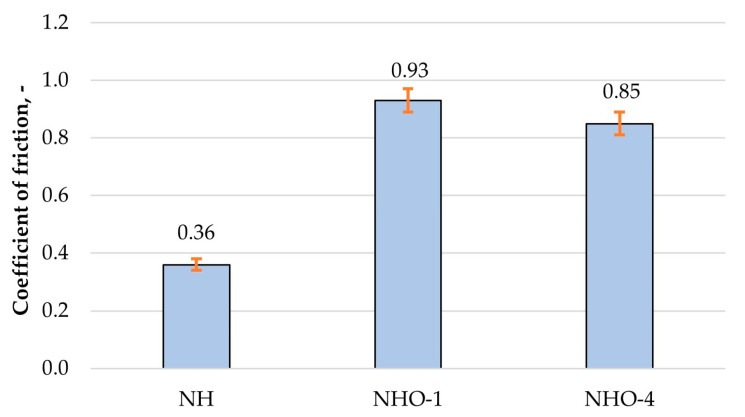
Friction coefficient.

The purpose of the tribological tests was to determine the friction coefficients of the friction pairs tested. Figure 11 shows graphs of average friction coefficients recorded during friction with lubrication by the artificial saliva solution.

Based on the results of tribological tests, it was determined that the stereometric parameters of the surface layer have a significant impact on tribological properties. The sample with the least developed surface, NH, exhibited the lowest friction coefficients, while the highest values were recorded for NHO-1. These were approximately 60% higher compared to the reference material. In the case of sample NHO-4, the recorded coefficients of friction were approximately 10% lower compared to NHO-1. This is most likely due to the better lubricating properties of this surface, related to the parameter Svk—i.e., the average depth of valleys below the core profile. The interaction of two elements moving relative to each other results in tribological wear, which largely depends on the stereometric properties of the triboelement surfaces.

### 3.5. Assessment of Surface Geometric Structure of Samples

**Figure 12:**
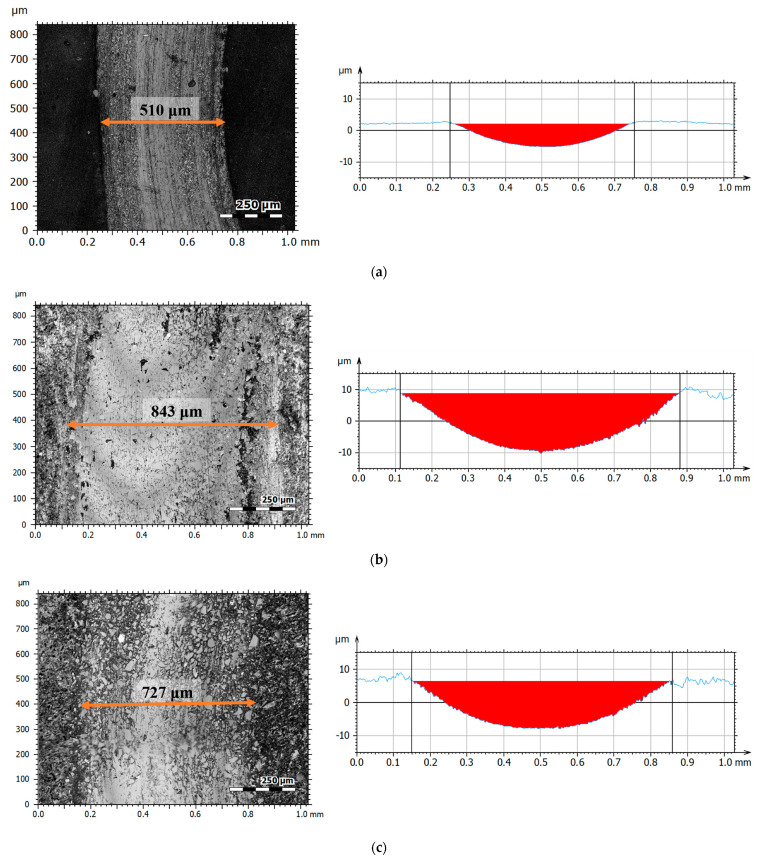
Wear microstructure and profiles: (**a**) NH, (**b**) NHO-1, (**c**) NHO-4.

**Figure 13:**
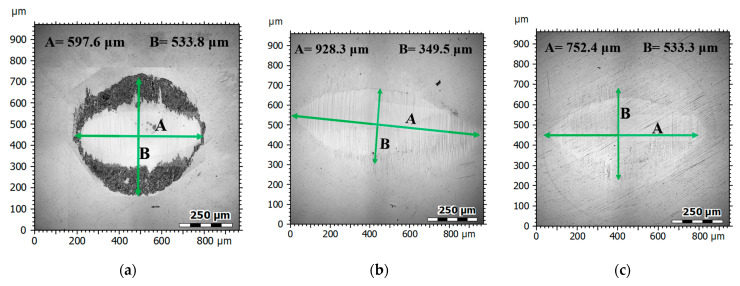
Ball wear track images: (**a**) NH, (**b**) NHO-1, (**c**) NHO-4.

**Figure 14:**
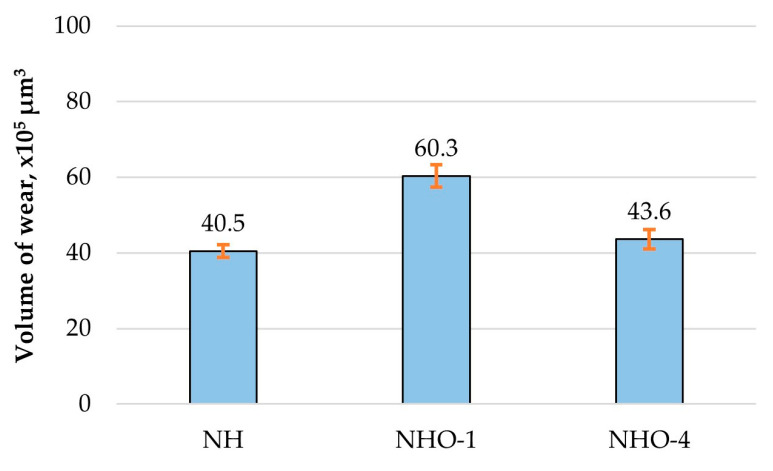
Volumetric wear of the samples.

Figure 12 presents microstructural images (a) and profiles (b) of the wear tracks (red area), Figure 13 shows photographs of the wear tracks on the balls, and Figure 14 presents the average wear track volume determined based on five series of measurements.

The microscopic examination results indicated that the greatest wear traces were recorded for the sample with a ceramic additive—NHO-1—and the least for the classic composite material—NH. The volumetric wear of sample NHO-4 was approximately 30% less compared to NHO-1 and resulted from the sample preparation method. In the case of NHO-1, the 3 mm thick material was polymerized as a whole, while in the case of NHO-4, each of the six layers, 0.5 mm thick, was polymerized separately, which improved its hardness and wear resistance.

## 4. Conclusions

The microscopic analysis showed that samples NHO-1 and NHO-4 had the most complex surface structures. Based on their material composition curves, these samples were predicted to exhibit the longest run-in time and best lubrication. The hardness tests confirmed that both the ceramic additive and the layer-by-layer polymerization method significantly improved the material’s mechanical properties. Compared to the standard composite, NHO-1 and NHO-4 were 30% and 43% harder, respectively. Moreover, building up the material in 0.5 mm layers provided better results than curing it in a single 3 mm block.

The results of contact angle measurements indicated the hydrophilic properties of the classic composite, while the samples with ceramic additives were hydrophobic. From a clinical standpoint, poorly wettable materials are more desirable, as an increased hydrophilicity of dental materials can accelerate bond degradation, increase the likelihood of microleakage between the patient’s tissues and the filling, and pose a potential risk of secondary caries development.

Based on the results of friction and wear tests, it was determined that the stereometric parameters of the surface layer have a significant impact on tribological properties. The classic composite exhibited the lowest friction coefficients, while the highest values were recorded for NHO-1. For the NHO-4 sample, the recorded values were approximately 10% lower compared to NHO-1, most likely due to the better lubricating properties of this surface.

The microscopic observations of the wear tracks after tribological tests showed that the classic composite—NH—exhibited the highest wear resistance. However, the material with ceramic additives—NHO-4—showed slightly worse results. The volumetric wear of this sample was only about 8% higher compared to NH.

The results suggest that further research is necessary to optimize polishing processes for improved surface quality. This includes determining optimal time and tools. Additionally, the influence of polishing pastes and composite wear under varying forces, temperatures, and coatings should be investigated to enhance the understanding of composite behavior in clinical settings, leading to the development of more durable and esthetically pleasing dental material.

## Figures and Tables

**Table 1:** Test materials were prepared according to the description below.

Parameters	Materials		
	NH	NHO-1	NHO-4
Sample	Classic composite	Bulk fill one layer	Bulk fill four layers
Type	Nano-hybrid	Nano-hybrid ormocer	Nano-hybrid ormocer
Matrix and filler	Based on silicone dioxide	Based on silicone dioxide	Based on silicone dioxide
Filler content (%by weight)	>82	84	84

**Table 2:** Mechanical and physicochemical properties of ZrO_2_ [28].

		Resistance To			
Vickers Hardness, GPa	Bending Strength, MPa	Thermal Shock, °C	Chemical Exposure	Thermal Expansion Coefficient, ×10^−6^/°C	Thermal Conductivity, W/(m × K)
13	1000	280	good	7.7	3

**Table 3:** Chemical composition of the lubricant—artificial saliva [29].

Artifical Saliva, g/dm^3^					
NaCl	KCl	CaCl_2_ × 2H_2_O	NaH_2_PO_4_ × 2H_2_O	Na_2_S × 9H_2_O	Urea
0.4	0.4	0.795	0.780	0.005	1.0

**Table 4:** The parameters of surface texture.

Parameter	NH		NHO-1		NHO-4	
	Mean	Stand. Dev.	Mean	Stand. Dev.	Mean	Stand. Dev.
Sq [μm]	0.21	0.02	1.58	0.15	1.53	0.15
Sv [μm]	1.66	0.16	4.58	0.45	3.84	0.45
Sp [μm]	1.81	0.19	4.91	0.23	4.45	0.23
Ssk	−0.05	0.27	0.69	0.25	0.68	0.25
Sku	4.27	0.45	2.49	0.34	2.44	0.34

## Data Availability

The original contributions presented in the study are included in the article, further inquiries can be directed to the corresponding author.
